# Insights into Variations in Chemical Profiles and Antioxidant Properties Among Different Parts of *Dalbergia odorifera*

**DOI:** 10.3390/plants14213279

**Published:** 2025-10-27

**Authors:** Yujie Xiao, Yakui Zhou, Jianhe Wei, Xiangsheng Zhao

**Affiliations:** 1 Institute of Medicinal Plant Development, Chinese Academy of Medical Sciences & Peking Union Medical College, Beijing 100193, China; xiaoyj0103@163.com (Y.X.); wjianh@263.net (J.W.); 2 Hainan Provincial Key Laboratory of Resources Conservation and Development of Southern Medicine, Hainan Branch of the Institute of Medicinal Plant Development, Chinese Academy of Medical Sciences & Peking Union Medical College, Haikou 570311, China; zhouyakui163@163.com

**Keywords:** *Dalbergia odorifera*, plant parts, mass spectrometry, metabolomics, antioxidant

## Abstract

*Dalbergia odorifera*, a rare and precious medicinal plant, has been used to treat cardio- and cerebrovascular diseases in China for thousands of years. *D. odorifera* heartwood (DOH) is usually considered to be the main part used for medicine, and other parts (leaf, DOL; flower, DOF; pod, DOP) of *D. odorifera* are neglected. In this paper, a systematic comparative study was conducted on phytochemicals and antioxidant properties of four parts of *D. odorifera.* A total of 72 volatile organic compounds (VOCs) and 820 nonvolatile organic compounds (NVOCs) were identified in four *D. odorifera* parts by GC-MS and UPLC-ESI-Q/TRAP-MS/MS, respectively. Differences in phytochemical profiles among the different parts were observed. DOH exhibited a significantly different level of *trans*-nerolidol and flavonoids compared to the other parts. Taking into account all the parameters measured, methanolic extracts of DOH, DOL, and DOF had good antioxidant activity, with the highest value in DOH, followed by DOL and DOF. Moreover, the strong antioxidant activity of the methanolic extract may be related to the flavonoid components. The results indicated that DOL and DOF also have potential for further development and utilization.

## 1. Introduction

*Dalbergia odorifera*, a semi-deciduous perennial tree from the *Leguminosae* family and the *Dalbergia* genus, boasts extraordinary economic and medicinal worth. Native to Hainan Province, it has been introduced for cultivation in Guangdong, Fujian, Guangxi, and Yunnan Provinces, China. Known for its premium quality timber, it is highly sought after for crafting high-end furniture, luxury musical instruments, and intricate handicrafts. Additionally, the dried heartwood of *D. odorifera* (DOH), derived from the stem and root, commonly known as Jiangxiang, is a traditional Chinese medicine widely used for stopping bleeding, relieving pain, treating cardiovascular diseases, and serving as a key ingredient in several medicinal formulations, such as Dieda Zhentong Plaster, Guan-Xin-Er-Hao Decoction, Qishen Yiqi Dropping Pill, and Guanxin Danshen Tablet [1]. However, due to the long maturation period required for heartwood formation and its multiple uses, wild resources of *D. odorifera* have been severely depleted, making it difficult to meet the growing demand for Jiangxiang in the traditional Chinese medicine market [2]. In contrast, the leaf, flower, and pod of *D. odorifera* (DOL, DOF, and DOP), which are abundant and renewable, are typically discarded as waste during timber harvesting [3]. Therefore, understanding the chemical compositions and pharmacological activities of these non-medicinal parts is crucial for gaining deeper insight into their potential for development and utilization.

Current research has primarily focused on the medicinal part, DOH, while studies that comprehensively characterize the chemical compositions and evaluate the biological activities of the non-medicinal parts (DOL, DOF, and DOP) remain limited. Flavonoids and essential oils are the key active components of DOH, exerting various pharmacological effects, including anti-inflammatory, anti-angina, anti-thrombotic, vasodilatory, anti-platelet, and anti-oxidative activities, among others [1,4,5]. The essential oil of DOH is mainly composed of *trans*-nerolidol and its oxides, which together account for more than 62% of the total content [6]. Both DOH essential oil and *trans*-nerolidol have been shown to protect against myocardial ischemia in isoproterenol-induced rats [7,8]. The primary flavonoids in DOH, such as sativanone, 3′-O-methylviolanone, butein, liquiritigenin, isoliquiritigenin, formononetin, medicarpin, butin, and dalbergin, exhibit a wide range of pharmacological activities [9]. Numerous studies have highlighted the cardiovascular-protective effects of many flavonoids found in *D. odorifera* [10,11,12,13,14,15]. However, research on the chemical composition and biological activities of DOL, DOF, and DOL remains limited. Therefore, it is important to conduct research on these non-medicinal parts and analyze their differences compared to DOH to make full use of *D. odorifera* resources.

Oxidative stress, an imbalance between the generation and clearance of reactive oxygen species (ROS), plays a crucial role in the pathogenesis of cardiovascular diseases [16]. Antioxidant activity is, therefore, a vital indicator of the quality evaluation of *D. odorifera*. DPPH (2,2-diphenyl-1-picrylhydrazyl) and ABTS (2,2′-Azinobis-(3-ethylbenzthiazoline-6-sulphonate)) free radical-scavenging assays, along with the ferric-reducing antioxidant power (FRAP) assay, were selected to evaluate the antioxidant activity of *D. odorifera* [17]. Consequently, the primary research priorities of this study are to analyze the chemical components and antioxidant activity of DOH, DOL, DOF, and DOP grown in Hainan Province, China. Firstly, metabolomics based on GC-MS and UPLC-MS/MS was performed to characterize the metabolic profiles, including VOCs and NVOCs, of different parts of *D. odorifera*. Differential metabolites (DMs) were screened using orthogonal partial least squares discriminant analysis (OPLS-DA) and then enriched using KEGG pathway enrichment analysis. Secondly, the contents of *trans*-nerolidol and 14 flavonoids were determined using GC-MS and UPLC-DAD. Thirdly, the antioxidative activity of each part was estimated using DPPH, ABTS, and FRAP methods. This study provides a reference for the development and utilization of *D. odorifera* resources.

## 2. Results and Discussion

### 2.1. VOCs Analysis of Four Parts of D. odorifera Based on GC-MS

**Figure 1:**
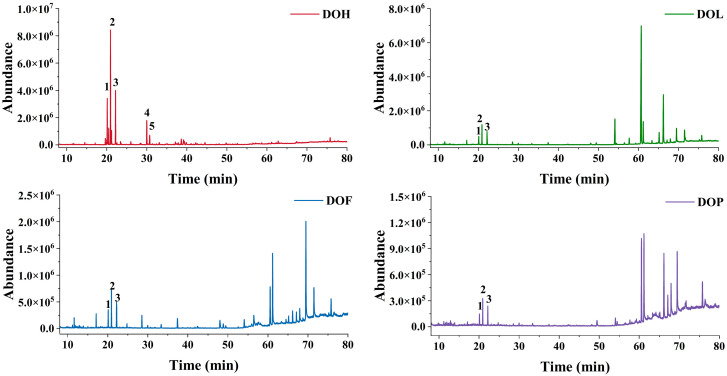
GC-MS total ion chromatograms (Peaks 1–5 represent nerolidol oxide I and II, *trans*-nerolidol, and nerolidol oxide III and IV, respectively).

The VOCs of *D. odorifera* were characterized by GC-MS, and the total ion chromatograms of the four parts are shown in Figure 1. The peak signal distribution of DOH is distinctly different from that of other parts, which implies that the VOC composition of DOH diverges significantly from the rest. In contrast, DOL, DOF, and DOP samples exhibit similar peak signal distributions, suggesting a similar VOC composition among them. Additionally, DOH exhibited a markedly higher peak signal than the other parts. Most of the VOCs were identified at level 2 according to the Metabolomics Standards Initiatives (MSI) via spectral matching against the NIST 17. *Trans*-nerolidol was identified by comparing the retention time and mass fragment patterns with a reference standard (MSI level 1). The relative molecular mass of *trans*-nerolidol is 222.37 (C_15_H_26_O), and its main feature fragment ions include *m/z* 69, 93, 107, 136, and 161 [18,19,20]. *Trans*-nerolidol oxide was identified by comparison with reported literature data (MSI level 2). The mass spectrometry fragment pathway of nerolidol oxide is presented in Figure S1. The peak order of *trans*-nerolidol and nerolidol oxide (I, II, III, and IV) is consistent with Tan C. et al. [21]. In total, there were 72 VOCs, of which 42, 22, 23, and 18 were detected in EA extracts from DOH, DOL, DOF, and DOP, respectively (Figure S2A). Profiling VOCs of four parts unveiled 4 shared compounds, with only 7, 8, and 4 compounds exclusive to DOL, DOF, and DOP, while 37 unique compounds were found in DOH.

Furthermore, the relative content of each compound was calculated via the peak area normalization method (%, Table S1). The DOH-EA was found to predominantly contain *trans*-nerolidol and four nerolidol oxide isomers (I-IV), with nerolidol oxide II being the most abundant compound. Notably, this differs from the findings of Wang et al., who reported *trans*-nerolidol as the dominant component in the ethanol-benzene extractive of DOH [22], a discrepancy potentially attributable to differences in the growth environment and storage conditions or extraction methods. In contrast, the main VOCs of DOL, DOF, and DOP are alkanes and fatty acids, consistent with the findings of Wu J et al. [23]. DOL-EA primarily contained tetracosane (41.06%) and heptacosane (11.50%). However, the main VOCs of DOL essential oil extracted via steam distillation are 2-methoxy-4-vinylphenol (21.73%), n-hexadecanoic acid (13.97%), and phenol (6.69%) [24], demonstrating that the type and content of VOCs are affected by the extraction method [25]. The main VOCs of DOF-EA are (Z,Z)-9,12-Octadecadienoic acid (17.99%), eicosane (9.95%), and n-hexadecanoic acid (9.63%). The main VOCs of DOP-EA are octadecanoic acid (17.47%), tetracosane (16.66%), eicosane (14.78%), and (Z,Z)-9,12-octadecadienoic acid (12.70%). VOCs of heartwood, the medicinal part of *D. odorifera*, markedly differ from those of other parts. In contrast, the DOL, DOF, and DOP exhibit greater similarity in their VOCs composition, as they are all rich in eicosane, (Z,Z)-9,12-octadecadienoic acid, 2,2′-methylenebis [6-(1,1-dimethylethyl)-4-methyl-phenol, etc. *Trans*-nerolidol and nerolidol oxide (I, II) were also detected in DOL, DOF, and DOP with low content (1.46~6.52%), from high to low in DOL, DOF, and DOP.

### 2.2. Discrepancies in VOCs of Different D. odorifera Parts

**Figure 2:**
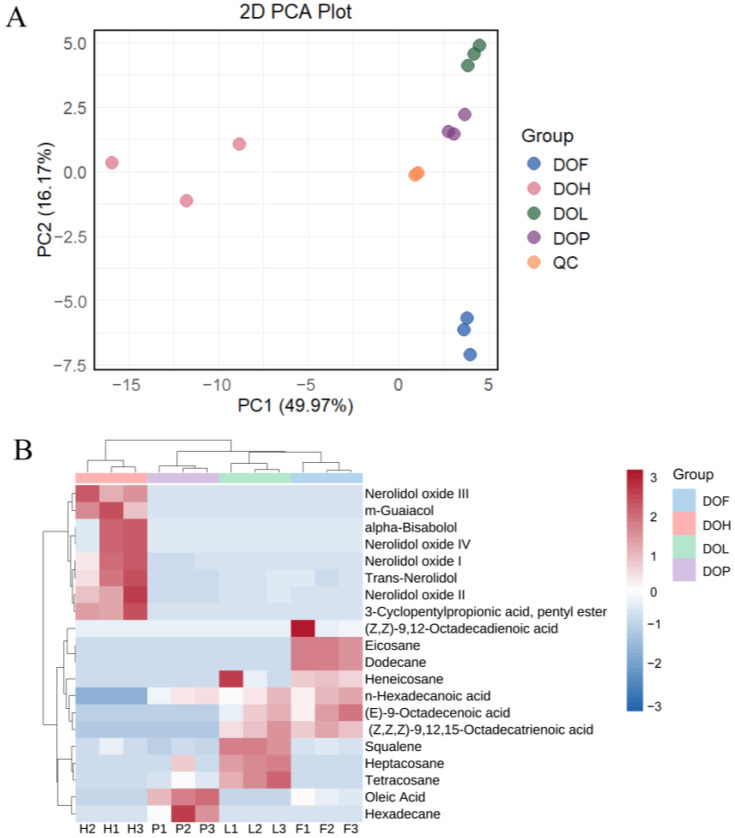
(**A**) Principal component analysis (PCA) of VOCs in DOH, DOL, DOF, and DOP. (**B**) Heatmaps of differential VOCs in DOH, DOL, DOF, and DOP.

After pretreatment of the metabolite data, an unsupervised principal component analysis (PCA) was performed to gain a preliminary understanding of the overall VOC differences between and within groups. As shown in Figure 2A, the first two principal components, PC1 and PC2, explained 49.97% and 16.17% of the total variations. QC samples are tightly clustered, with their coordinates consistently positioned near the origin, demonstrating the high robustness and reliability of the analytical platform. Compared with samples of other parts, DOH samples are more dispersed in the PCA score plot, attributable to their higher diversity in VOCs (more variables) [26]. In the PCA score plot, DOL, DOF, and DOP showed close proximity to each other, collectively occupying the positive axis of the first principal component (PC1). Instead, DOH formed a separate cluster positioned at the negative PC1 axis. This indicates that the similarity among DOL, DOF, and DOP is greater than DOH, consistent with the previous findings. PC1 reflects the variation between DOH and other parts, while the second principal component (PC2) reflects the variation among DOsL, DOF, and DOP.

Orthogonal partial least squares-discriminant analysis (OPLS-DA) was conducted to maximize inter-group separation and screen differential VOCs among different parts. The model quality was evaluated using three parameters: Q^2^ reflecting the model’s predictive capability through cross-validation, and R^2^X/R^2^Y representing the explained variance of X/Y matrices, respectively. The metrics of recognition ability (R^2^X and R^2^Y) and predictive ability (Q^2^) were both > 0.9, with R^2^Y and Q^2^ values close to 1, which indicated that the OPLS-DA model was stable and reliable for this dataset. OPLS-DA score scatter plots revealed differences among DOH, DOL, DOF, and DOP based on intergroup comparisons. Consequently, there was remarkable discrimination in VOCs among different *D. odorifera* parts. A total of 200 random permutation tests were verified in order to avoid overfitting. The result revealed that the values of R^2^ were close to 1 (greater than 0.99), suggesting that the OPLS-DA model was accurate and valid.

The differential VOCs were subsequently identified according to VIP > 1 and fold changes ≥2 or ≤0.5. A total of 20 differential VOCs were filtered from four *D. odorifera* parts. These differential VOCs mainly included *trans*-nerolidol, nerolidol oxides, aliphatic hydrocarbons, fatty acids, and some other compounds. The variations of these 20 DMs could be intuitively visualized by a heat map combined with two-way hierarchical clustering analysis (HCA) (Figure 2B). DOL, DOF, and DOP samples gathered together, consistent with their obvious classifications in the PCA model. The results indicated significant metabolic differences in different *D. odorifera* parts. It was not only unique compounds that caused these differences, but also variations in shared compounds. In general, eight volatile metabolites were observed with higher levels in DOH, seven in DOL, seven in DOF, and three in DOP. *Trans*-nerolidol and nerolidol oxides were previously reported as important active ingredients in DOH [7]. *Trans*-nerolidol and nerolidol oxides were, therefore, selected as key differential VOCs in DOH to distinguish from other *D. odorifera* parts.

### 2.3. NVOCs Analysis of Four Parts of D. odorifera Based on UPLC-MS/MS

Widely targeted metabolomics enables a comprehensive and systematic comparison of different samples through the simultaneous detection of multiple small molecular metabolites. The comprehensive metabolomics analysis based on UPLC-MS/MS characterized or tentatively identified 820 NVOCs from the four parts of *D. odorifera* in both positive and negative ion modes (Table S2), including 794 flavonoids and 26 tannins. Flavonoids play crucial roles in plant development and growth [27], making them a significant factor in *D. odorifera*’s metabolite composition. In total, 565, 650, 619, and 570 NVOCs were identified in DOH, DOL, DOF, and DOP, respectively (Figure S2B). Among them, 356 shared NVOCs were detected across all these parts, while 102, 16, 4, and 12 unique compounds were exclusively identified in DOH, DOL, DOF, and DOP, respectively. Therefore, heartwood, the medicinal part of *D. odorifera*, exhibited the most unique metabolites. In addition, DOL is the most diverse part of *D. odorifera* with the most NVOCs detected.

**Figure 3:**
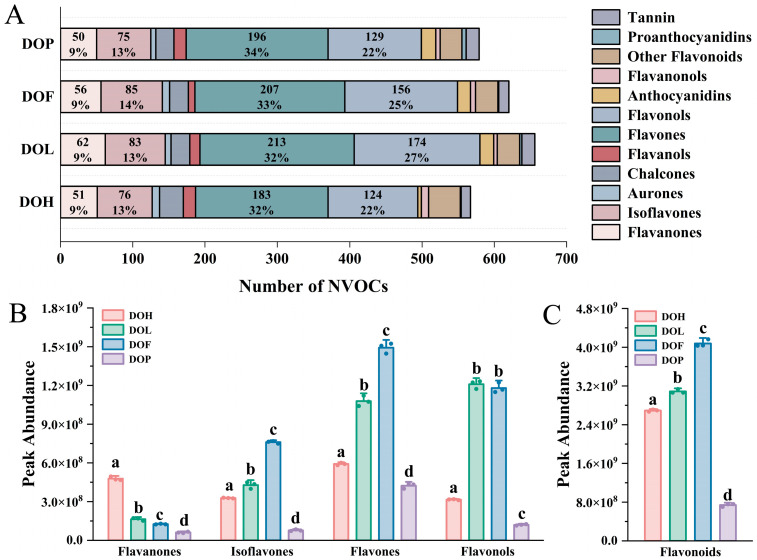
(**A**) Number of NVOCs in different parts. (**B**) Raw peak abundance of flavanones, isoflavones, flavones, and flavonols. (**C**) Total peak abundance of flavonoids. Error bars represent standard deviation (SD) of three independent experiments (*n* = 3). Different letters indicate significant differences among groups (*p* < 0.05, one-way ANOVA followed by Tukey’s post hoc test).

Flavonoids constitute the most abundant and functionally diverse class of secondary metabolites among *D. odorifera* polyphenols and serve as important active indicators for evaluating *D. odorifera* quality [1]. Therefore, the composition of flavonoids in different parts was further analyzed. The class II distribution of flavonoids in each part is extremely similar (Figure 3A). Flavones and flavonols account for the highest proportion (more than 50%), followed by isoflavonoids (13–14%) and flavanones (9%). To explore the discrepancies in the relative content of different chemical classes of flavonoids among four different *D. odorifera* parts, a statistical analysis based on the peak abundance of the identified flavonoids was conducted. Flavanones, isoflavones, flavones, and flavonols are the predominant flavonoid classes in *D. odorifera*, exhibiting the broadest distribution and highest abundance throughout all four parts. The total peak abundance distribution of flavonoids in the analyzed samples is presented in Figure 3C, and that of the four identified phytochemical classes is presented in Figure 3B. Comparative analysis revealed distinct distribution patterns of flavonoid classes across different parts. DOL and DOF exhibited a significantly higher accumulation of isoflavones and flavones compared to DOH and DOP (*p* < 0.05). In contrast, DOH demonstrated a predominant accumulation of flavanones, showing statistically significant differences from other parts (*p* < 0.01). Notably, DOP contained the lowest total flavonoid content among all analyzed plant parts (*p* < 0.001). These results showed that the number and content of chemical components in the DOP are both lower than in the other parts of *D. odorifera*.

### 2.4. Discrepancies in NVOCs of Different D. odorifera Parts

**Figure 4:**
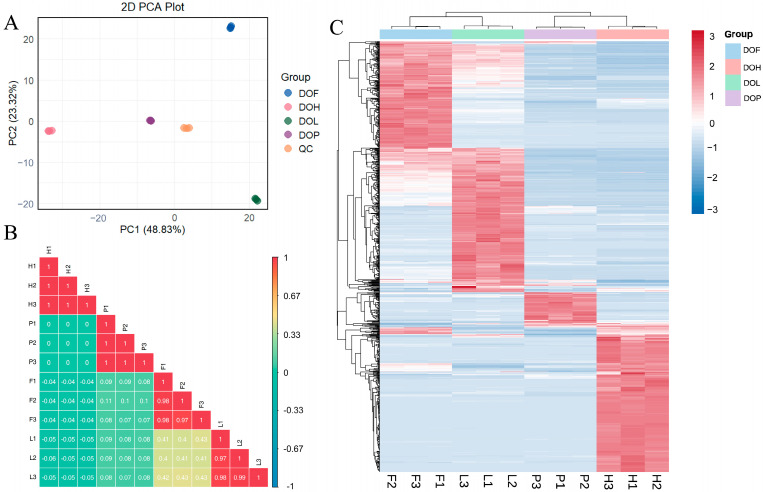
Metabolomics analysis of NVOCs in different *D. odorifera* parts based on UPLC-MS/MS. (**A**) Principal component analysis (PCA) of NVOCs in different sample groups. (**B**) Correlation analysis of different samples. (**C**) Heatmaps of differential NVOCs in different sample groups.

PCA was used to assess the variability in NVOCs of different *D. odorifera* parts. The QC samples were tightly clustered at the center of the PCA score plot, indicating desirable stability and reproducibility of the widely targeted metabolomics based on UPLC-ESI-Q TRAP-MS/MS (Figure 4A). The PCA model effectively captured distinct metabolic profiles, accurately differentiating the characteristic metabolic variations among each part. All *D. odorifera* samples could be differentiated along the horizontal or vertical axis. The correlation analysis (Figure 4B) indicated high repeatability within the DOH, DOL, DOF, and DOP groups, suggesting reliable sample consistency. In contrast, the correlation between the different groups was low, highlighting significant variations in metabolite profiles between DOH, DOL, DOF, and DOP. Notably, DOL and DOF showed higher correlation coefficients (*r* = 0.4) compared to other tissue pairs, suggesting a closer metabolic relationship between these two tissue types.

OPLS-DA can obtain a relatively desirable discrimination between DOH, DOL, DOF, and DOP, with R^2^Y and Q^2^ cross-validation greater than 0.9. Differential NVOCs were filtered according to VIP > 1 and fold changes ≥2 or ≤0.5. Overall, 678, 682, 568, 458, 556, and 536 differential NVOCs were screened in the DOH_vs_DOL, DOH_vs_DOF, DOH_vs_DOP, DOL_vs_DOF, DOL_vs_DOP, and DOF_vs_DOP comparisons. Heatmaps combined with HCA were used to visualize the distribution patterns of differential NVOCs (Figure 4C). HCA results suggested that 273 compounds had higher levels in DOH, 273 had higher levels in DOL, 201 had higher levels in DOF, and 66 had higher levels in DOP. In the heatmap, samples could be clearly divided into four groups, indicating significant differences in metabolite composition among different *D. odorifera* parts. The distributions suggested that DOH and DOL exhibited the highest DMs richness, followed by DOF, with DOP containing the fewest metabolites. The main classes of DMs are flavonoids (96.8%), indicating that the tissue differences in *D. odorifera* NVOCs were largely concentrated in this category.

### 2.5. KEGG Pathway Enrichment Analysis of DMs

**Figure 5:**
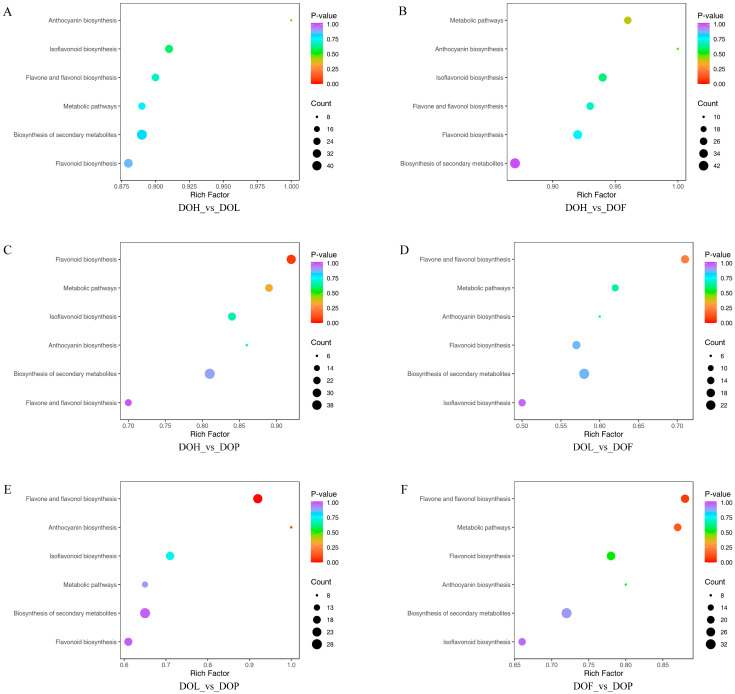
KEGG pathway enrichment analysis of DMs in four *D. odorifera* parts. (**A**–**F**) representing the DMs enrichment pathways of DOH_vs_DOL, DOH_vs_DOF, DOH_vs_DOP, DOL_vs_DOF, DOL_vs_DOP, and DOF_vs_DOP, respectively.

The interconversion of different metabolites supports diverse organismal functions, and KEGG-based metabolic analysis facilitates deeper investigation of these biological processes [28]. To further identify the differences in the metabolic pathways among the four *D. odorifera* parts, 813 DMs were mapped to the KEGG database. The KEGG pathway enrichment analysis indicated that 103 DMs were annotated in the 6 metabolic pathways, including metabolic pathways, the biosynthesis of secondary metabolites, flavonoid biosynthesis, flavone and flavonol biosynthesis, anthocyanin biosynthesis, and isoflavonoid biosynthesis. Among them, three pathways (flavone and flavonol, flavonoid, and anthocyanin biosynthesis) were identified as the most enriched according to *p* value and impact value, making important contributions to the formation of DMs. The results showed that the most abundant metabolite enrichment was the biosynthesis of secondary metabolites, and the largest enrichment factor was anthocyanin biosynthesis. DMs were mainly enriched in the flavone and flavonol biosynthesis pathway in the comparison groups DOL_vs_DOF and DOF_vs_DOP (Figure 5D,F). Additionally, DMs were mainly enriched in the flavonoid biosynthesis pathway in the DOH_vs_DOP comparison group (Figure 5C). In the comparison group DOL_vs_DOP, DMs were mainly enriched in the anthocyanin, flavone, and flavonol biosynthesis pathway (Figure 5E). However, in the comparison groups DOH_vs_DOL and DOH_vs_DOF, there was no significant enrichment observed in the flavonoid synthesis pathway (Figure 5A,B). Flavonoid metabolism was also one of the important pathways of plant secondary metabolism, playing a critical role in the plant growth and development process [29]. The biosynthesis of flavonoids occurs at the junction of the shikimate pathway (polyketide pathway) and the acetate pathway, among which naringenin is the common precursor of most intermediate metabolites and end products [27]. All significant metabolic pathways were related to flavonoid metabolism, and their related substances were analyzed in detail. A total of 103 DMs associated with biosynthetic pathways are summarized in Table S3. Some DMs were involved in more than one metabolic pathway. For example, isoliquiritigenin, eriodictyol, taxifolin, and naringenin chalcone are involved in flavonoid biosynthesis, metabolic pathways, and the biosynthesis of secondary metabolites. Vestitol, calycosin, 3,9-dihydroxypterocarpan, and 4′,6,7-trihydroxyisoflavone were involved in isoflavonoid biosynthesis and the biosynthesis of secondary metabolites.

### 2.6. Quantitative Analysis of Main Compounds in Different D. odorifera Parts

#### 2.6.1. Contents of Trans-Nerolidol Evaluated by GC-MS

**Figure 6:**
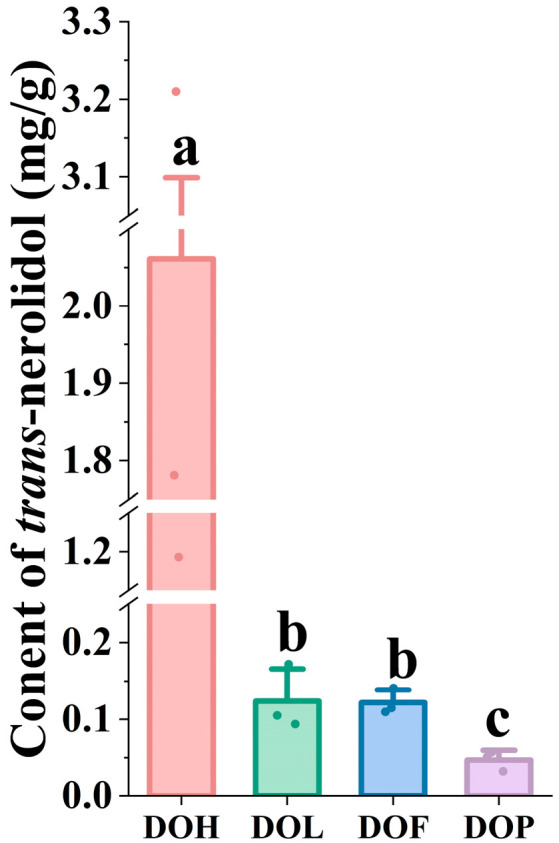
The content of *trans*-nerolidol in different *D. odorifera* parts. Error bars represent standard deviation (SD) of three independent experiments (*n* = 3). Different letters indicate significant differences among groups (*p* < 0.05, one-way ANOVA followed by Tukey’s post hoc test).

Nerolidol (3,7,11-trimethyl-1,6,10-dodecatrien-3-ol) is a sesquiterpene alcohol with multiple pharmacological activities, widely occurring in plant essential oils [25,30]. Particularly, *trans*-nerolidol is the primary volatile component (45.23–69.13%) of essential oils in the DOH [31], and *trans*-nerolidol and nerolidol oxides reach a level of more than 62% [6]. *Trans*-nerolidol was considered a distinctive compound in heartwood possessing diversified pharmacological effects [23]. As the material basis for protecting against myocardial ischemia, nerolidol and its oxides are significant indicators for identifying and evaluating the quality of *D. odorifera*. Therefore, the contents of *trans*-nerolidol were determined using GC-MS to elucidate differences in DOL, DOF, and DOP compared to DOH. To efficiently analyze *trans*-nerolidol, the sample preparation methods and instrumental conditions were optimized. Representative SIM chromatograms and the quantitative results are shown in Figure S3 and Figure 6. Two nerolidol oxides (i.e., III and IV) could not be detected in DOL, DOF, and DOP (<LOD). Compared with nerolidol oxide (III, IV), which were only detected in specific samples (DOH), *trans*-nerolidol and nerolidol oxide (I, II) were identified as the predominant forms in *D. odorifera*. One-way ANOVA indicated that *trans*-nerolidol, nerolidol oxide I, and nerolidol oxide II showed highly significant (*p* < 0.05) variation in different *D. odorifera* parts. Quantitative analysis revealed *trans*-nerolidol concentration of 1.193 to 3.210 mg/g in DOH, similar to the study by Tan C et al. [21]. In contrast to DOH, the other parts exhibited significantly lower *trans*-nerolidol content, ranging from 0.094 to 0.172 mg/g in DOL, 0.110 to 0.141 mg/g in DOF, and 0.032 to 0.057 mg/g in DOP.

#### 2.6.2. Contents of Fourteen Flavonoids Evaluated by UPLC-DAD

Flavonoids are the dominant compounds in DOH [9,32]. Meanwhile, numerous studies have demonstrated that flavonoids in *D. odorifera* possess various biological activities, such as antioxidant, anti-inflammatory, antibacterial, antitumor, and cardiovascular protection. Therefore, flavonoids could be considered as marker compounds to evaluate the quality of *D. odorifera*. The contents of 14 flavonoids, namely, sativanone, liquiritigenin, 3′,4′,7-trihydroxyisoflavone, butin, daidzein, luteolin, naringenin, tectorigenin, alpinetin, isoliquiritigenin, formononetin, 3′-O-methylviolanone, pinocembrin, and biochanin A, were determined using the UPLC-DAD method to illuminate their content differences in DOL, DOF, and DOP compared to DOH. In our previous work, the extraction conditions (solvent, volume, and time) and UPLC conditions (column and mobile phase) for the determination of heartwood flavonoids were optimized [9]. In this work, the column temperature, flow rate, and elution procedure were explored. To ensure that the established UPLC-DAD method could be applied to analyze the main flavonoids in *D. odorifera*, the linearity, LOD, LOQ, precision, repeatability, stability, and recovery were validated (Tables S4–S6).

**Figure 7:**
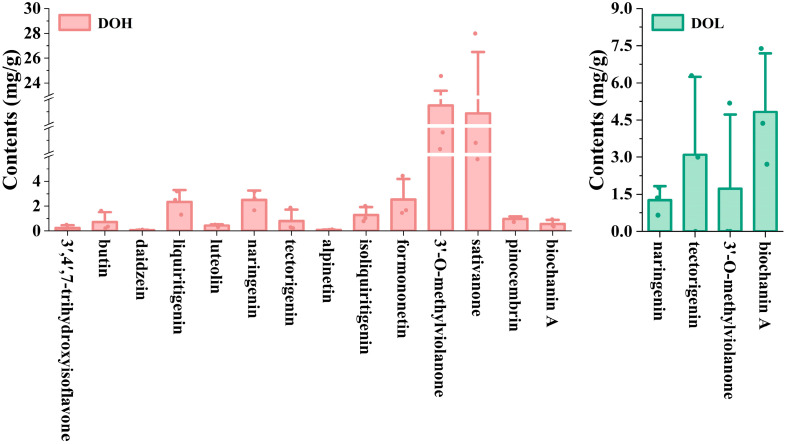
The contents of 14 flavonoids in different *D. odorifera* parts. Error bars represent standard deviation (SD) of three independent experiments (*n* = 3).

The validated UPLC-DAD method was applied to determine the 14 selected flavonoids in different *D. odorifera* parts. Representative chromatograms and the quantitative results are shown in Figure S4 and Figure 7. The contents of the 14 analytes varied in different *D. odorifera* parts. Sativanone and 3′-O-methylviolanone were the main compounds in DOH. The content of sativanone in all DOH batches ranged from 5.782 to 27.991 mg/g (4.84-fold variation), and that of 3′-O-methylviolanone ranged from 10.293 to 24.561 mg/g (2.39-fold variation), similar to those in previous studies [9,32]. DOL contains four of the fourteen analytes (naringenin, tectorigenin, 3′-O-methylviolanone, and biochanin A), whereas none of these analytes were detected in the DOF or DOP. Among them, tectorigenin and biochanin A were previously isolated from leaves [33]. In addition, the contents of tectorigenin and 3′-O-methylviolanone in partial DOL samples were lower than the LOQ. DOL contains significantly lower levels of naringenin, tectorigenin, and 3′-O-methylviolanone compared to DOH, whereas the biochanin A content is significantly higher than that in DOH (*p* < 0.01). Zhou S et al. found that the contents of biochanin A and genistein in DOL from different producing areas ranged from 0.45 to 38.43 μg/g and 0.58 to 79.76 μg/g [34]. The variations in flavonoid levels among different samples from the same part may result from differences in geographical conditions, tree ages, plant origins, and storage conditions.

### 2.7. Antioxidant Activity Evaluation of Four Plant Parts of D. odorifera

**Figure 8:**
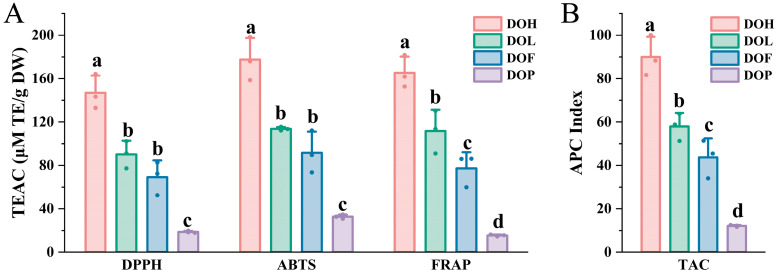
Evaluation of total antioxidant capacity (TAC) of four *D. odorifera* parts. (**A**) TEAC values of different *D. odorifera* parts determined via DPPH, ABTS, and FRAP methods. (**B**) Total antioxidant capacity, expressed as APC index, of different *D. odorifera* parts. Error bars represent standard deviation (SD) of three independent experiments (*n* = 3). Different letters indicate significant differences among groups (*p* < 0.05, one-way ANOVA followed by Tukey’s post hoc test).

*D. odorifera* has been demonstrated to provide diverse health benefits, which are closely related to the antioxidant activity of its flavonoid and polyphenol metabolites [11,35]. To comprehensively assess the antioxidant activity of all *D. odorifera* samples, DPPH, ABTS radical-scavenging assay and FRAP assay were conducted (Figure 8A). The trolox equivalent antioxidant capacity (TEAC) values of DPPH, ABTS, and FRAP assay ranged from 18.01 to 164.29 μM TE/g DW (9.12-fold variation), 30.91 to 198.19 μM TE/g DW (6.41-fold variation), and 14.49 to 130.44 μM TE/g DW (9.00-fold variation), respectively. This phenomenon can be attributed to the distinct reaction mechanisms between various sample components and different free radical species. The antioxidant activities of *D. odorifera* varied significantly between different *D. odorifera* parts (*p* < 0.05), with similar trends emerging across all three methods. In ABTS^+^ and DPPH assays, DOH demonstrated the highest antioxidant capacity, followed by DOL and DOF, whereas DOP had the lowest antioxidant capacity. The FRAP experiments further confirmed that the antioxidant capacity was the highest in DOH, followed by DOL and DOF, with DOP showing the lowest levels; thus, it can be concluded that DOH possessed the strongest antioxidant activity. Flavonoids are known for their strong antioxidant properties [27]. The antioxidant activity of *D. odorifera* may be attributed to its flavonoid content. Generally, DOP exhibited the lowest antioxidant capacity among all parts, consistent with its low flavonoid content.

The reaction mechanisms of the three methods are different, resulting in some discrepancies in the results [17,36]. Therefore, an overall APC index was calculated for each sample according to the method outlined by Seeram et al. [37]. Significant differences in the APC index were observed among four *D. odorifera* parts (Figure 8B). DOH2 exhibited the highest APC index among all the samples, indicating the strongest antioxidant capacity. The results revealed that DOH, DOL, and DOF exhibited potent antioxidant properties, with DOH demonstrating the highest activity, whereas DOP showed significantly lower values (*p* < 0.001). These findings indicate that DOH, DOL, and DOF are promising sources of natural antioxidants. These insights offer a solid scientific foundation for the comprehensive development of natural antioxidants based on DOH, DOL, and DOF. DOL and DOF have potential medicinal value, as it is reported that DOL could exert a hypolipidemic effect [38] and attenuate cerebral ischemia–reperfusion injury [39].

## 3. Materials and Methods

### 3.1. Materials and Chemical Reagents

*Trans*-nerolidol was purchased from Shanghai Yuanye Bio-Technology Co., Ltd. (Shanghai, China). Sativanone and 3′-O-methylviolanone were purchased from Wuhan Tianzhi Biotechnology Co., Ltd. (Wuhan, China). Liquiritigenin, 3′,4′,7-trihydroxyisoflavone, daidzein, luteolin, tectorigenin, alpinetin, and isoliquiritigenin were purchased from Chengdu Chroma-Biotechnology Co., Ltd. (Chengdu, China). Formononetin, naringenin, pinocembrin, and biochanin A were purchased from Sichuan Wictory Biological Technology Co., Ltd. (Chengdu, China). The purities of the above-mentioned components were greater than 98%. HPLC-grade methanol, acetonitrile, acetic acid, and ethyl acetate were purchased from Thermo Fisher Scientific Inc. Co., Ltd. (Waltham, MA, USA).

The four *D. odorifera* parts (heartwood, leaf, flower, and pod) were collected from Haikou, Wanning, and Dongfang, Hainan Province, China. Voucher specimens (No. 469027-LD-021) were deposited in the Resource Center for Chinese Materia Medica, Hainan Branch Institute of Medicinal Plant Development, Chinese Academy of Medical Sciences & Peking Union Medical College (Haikou, China). The samples were collected under appropriate permits and authenticated by Professor Xiangsheng Zhao (Hainan Branch of the Institute of Medicinal Plant Development, Peking Union Medical College, Chinese Academy of Medical Science). The dry crude materials were ground into a fine powder prior to analysis. The heartwood, leaf, flower, and pod of *D. odorifera* were designated as DOH, DOL, DOF, and DOP, respectively.

### 3.2. Determination of Fourteen Flavonoid Compounds by UPLC-DAD

Sample powder (200 mg) was extracted via ultrasonication with 25 mL 70% (*v/v*) methanol solution at 40 °C for 45 min. The solution was cooled to room temperature and adjusted to the original weight with methanol solution, then centrifuged at 8000 r/min for 5 min. Subsequently, the supernatant was filtrated through a 0.22 μm membrane filter prior to UPLC-DAD analysis. Each sample was injected in triplicate.

Quantitative analysis of 14 flavonoid compounds using Agilent 1290 Infinity II series UPLC system (Agilent Technologies, Santa Clara, CA, USA) coupled with Diode Array Detector (DAD). A Waters Xbridge HSS T_3_ chromatogram column (100 mm × 2.1 mm, 1.8 μm) with solvent A (0.3% acetic acid aqueous solution) and solvent B (acetonitrile) as the mobile phases was prepared for UPLC separation. The gradient elution system was as follows: 0–3 min, 5–28% B; 3–9 min, 28–34% B; 9–11 min, 34–55% B, held at 55% B for 2 min; 13–18 min, 55–80% B; 18-19 min, 80–5% B. The flow rate of 0.3 mL/min and the column temperature were set to 40 °C. The injection volume was 5 μL. The detection was recorded at 275 nm and 360 nm.

### 3.3. Determination of Trans-Nerolidol and Metabolomics Analysis of VOCs by GC–MS

Sample powder (200 mg) was extracted via ultrasonication with 4 mL of ethyl acetate (EA) at 70 °C for 80 min. The solution was cooled to room temperature and adjusted to the original weight with EA, then centrifuged at 8000 r/min for 5 min. The obtained supernatant was filtered using a 0.22 μm membrane filter. Three QC samples were prepared by mixing equal aliquots of all biological samples from each batch, and these QC samples were analyzed periodically to evaluate the reproducibility and stability of the analytical system. Each sample was injected in triplicate as technical replicates.

An Agilent 7890A GC equipped with a 5975C quadrupole mass spectrometer (Agilent, California, USA) was employed to qualitatively and quantitatively analyze the VOCs and *trans*-nerolidol. Chromatography was performed on an HP-INNOWax column (30 m × 0.25 mm, 0.25 μm, Agilent, USA) with a flow rate of carried gas (helium) at a constant flow rate of 1.0 mL·min^−1^. The injection volume was 1 μL using split mode (10:1). The oven temperature gradient was programed as follows: initial temperature of 100 °C, held for 2 min; increased to 160 °C at 3 °C/min; increased to 190 °C at 1 °C/min; increased to 220 °C at 5 °C/min; increased to 250 °C at 2 °C/min, held for 7 min. The MS was carried out in the electron ionization mode with an electron energy of 70 eV. A solvent delay of 8 min was used to prevent damage to the ion source filament. The temperature of the injector, transfer line, and ion source was set at 240, 250, and 230 °C, respectively. The full scan mode was employed with the *m/z* range of 50–600 for qualitative analysis. The SIM mode was used for quantitative analysis of *trans*-nerolidol. Five ions (*m/z*: 69, 93, 107, 136, 161) were selected for *trans*-nerolidol, among which *m/z* 69 was used for quantitation.

### 3.4. Metabolomics Analysis of NVOCs by UPLC-ESI-Q TRAP-MS/MS

The *D. odorifera* samples were freeze-dried in a vacuum for 63 h and then ground into powder. Subsequently, the non-volatile compounds in each sample (50 mg) were extracted with 1200 μL of 70% methanol (with 0.25 mg/mL of L-2-chlorophenylalanine as the internal standard) by vortexing for 30 s every 30 min, repeated 6 times in total. Then, the sample extracts were centrifuged at 12,000 r/min for 3 min, and the supernatants were filtered using 0.2 μm polytetrafluoroethylene filters for LC-MS/MS analysis. The quality control (QC) samples were prepared by mixing an equal aliquot of the supernatants to evaluate the reproducibility and stability of the LC-MS/MS method.

A UPLC system (ExionLC™ AD, SCIEX, Concord, ON, Canada) with an Agilent SB-C18 column (1.8 µm, 2.1 mm × 100 mm; Agilent Corporation, California, CA, USA) coupled to a Quadrupole-Orbitrap mass spectrometer was used for the LC-MS/MS analysis. The mobile phase consisted of water (containing 0.1% formic acid, A) and acetonitrile (containing 0.1% formic acid). The gradient program was as follows: 0–9 min, 5–95% B, hold on 1 min, 11.1 min, 5% B, hold on 2.9 min. The column temperature was maintained at 40 °C, and the injection volume was 2 μL. Three QC samples were prepared by mixing equal aliquots of all biological samples from each batch. Each sample was injected in triplicate.

Widely targeted metabolomic analysis of NVOCs was performed using a UPLC system (ExionLC™ AD) coupled with a triple quadrupole linear ion trap mass spectrometer (QTRAP 6500+, AB SCIEX, Concord, ON, Canada) equipped with an electrospray ionization (ESI) source. The instrument was operated in both positive and negative ion modes under multiple reaction monitoring (MRM) conditions. The MS parameters were as follows: source temperature of 500 °C; ion spray voltage (IS) of 5500 V (positive ion mode)/−4500 V (negative ion mode); ion source gas I (GSI), gas II (GSII), and curtain gas (CUR) set to 50, 60, and 25 psi, respectively; and high collision-activated dissociation (CAD). The declustering potential (DP) and collision energy (CE) for individual MRM transitions were determined with further DP and CE optimization. A specific set of MRM transitions was monitored for each period according to the metabolites eluted within this period.

### 3.5. Multivariate Statistical Analysis

After obtaining the raw mass spectrometry (MS) data, subsequent processing was performed using Analyst 1.6.3 for data pre-processing, including peak detection, extraction, alignment, normalization, and integration. The local database was used for metabolite information matching. Following this pre-processing, an evaluation was performed: If more than 75% of the potential peaks in the QC samples had a relative standard deviation (RSD) of less than 30%, this indicates robust stability of the detection system and confirms the reliability of the generated data. Based on the local metabolic database and multiple reaction monitoring (MRM) mode, the metabolites of each sample were qualitatively analyzed by MS.

Subsequently, R version 4.1.2 were applied for multivariate data analysis, including principal component analysis (PCA), hierarchical cluster analysis (HCA), and orthogonal partial least-squares discriminant analysis (OPLS-DA). Unsupervised PCA was performed using the statistics function prcomp after the data were unit-variance scaled. HCA was performed using the R package ComplexHeatmap. The HCA results of samples and metabolites were presented as heatmaps with dendrograms, while Pearson correlation coefficients (PCC) between samples were calculated by the cor function in R and presented as only heatmaps. OPLS-DA was performed using the R package MetaboAnalystR. The results of OPLS-DA were presented as score plots, S-plot, and permutation plots.

### 3.6. Determination of Antioxidant Activity

A total of 200 mg of powder of each sample was accurately weighed and extracted via ultrasonication with 25 mL of 70% methanol for 45 min. The solution was cooled to room temperature and adjusted to the original weight with 70% methanol, then centrifuged to obtain the supernatant for the antioxidant activity assay. Each experiment was repeated three times in parallel.

The DPPH radical scavenging assay was conducted according to Thaipong et al. [40] with some modifications. A working solution of 0.04 mg·mL^−1^ DPPH was prepared in anhydrous ethanol before use. Subsequently, 10 μL of the diluted sample was mixed with 190 μL of DPPH working solution. The mixture was then incubated at 37 °C in the dark for 2 h, and the absorbance was measured at λ = 517 nm. Then, 70% methanol was used as a blank. Results obtained were reported as μM Trolox equivalent (TE)/g DW. Additional dilution was needed if the value measured was over the linear range of the standard curve.

The ABTS radical-scavenging assay was performed according to the procedure described in the Total Antioxidant Capacity Assay Kit with ABTS (Beyotime Institute of Biotechnology). Equal amounts of ABTS solution and oxidant solution were mixed well and kept away from light for 12 h. Before use, dilute with 80% ethanol until its absorbance at 734 nm is 0.700 ± 0.05. The diluted samples (10 μL, 5 mg/mL) and ABTS^+^ radical (200 μL) were reacted at 37 °C in the dark for 5 min. The absorbance was detected at 734 nm, and the μM TE/g DW was calculated.

The ferric-reducing antioxidant power (FRAP) assay was performed according to the procedure described in the Total Antioxidant Capacity Assay Kit with FRAP (Beyotime Institute of Biotechnology). TPTZ diluent, TPTZ solution, and detection buffer solution were mixed in a ratio of 10:1:1 to prepare the FRAP working solution. Subsequently, the diluted samples (10 μL, 5 mg/mL) and FRAP work solution (180 μL) were reacted at 37 °C in the dark for 7 min. The absorbance was detected at 593 nm, and the μM TE/g DW was calculated.

Antioxidant capacity values were determined in three replicates for each sample tested, and the mean values ± standard deviation (SD) were reported. An overall antioxidant potency composite (APC) index was determined by assigning all assays an equal weight, assigning an index value of 100 to the best score for each test, and then calculating an index score for all other samples within the test as follows: antioxidant index score = [(sample score/best score) × 100]; the average of all three tests for each sample was then taken for the APC index. All assays were given equal weight, and an overall mean index value was calculated on a normalized basis for each sample.

### 3.7. Data Analysis

Statistical analyses were performed using SPSS 26.0 software (IBM Corp., Armonk, NY, USA). Data are presented as mean ± standard deviation (SD) from three independent experiments (*n* = 3). The normality of data distribution was verified using the Shapiro–Wilk test. One-way analysis of variance (ANOVA) was applied to assess differences among groups, followed by Tukey’s multiple comparison test for post hoc analysis. Differences were considered statistically significant at *p* < 0.05. Different letters above the bars indicate significant differences among groups (*p* < 0.05, one-way ANOVA with Tukey’s post hoc test).

## 4. Conclusions

In this study, the UPLC-DAD, GC-MS, and UPLC-MS/MS analysis methods were used to comprehensively investigate the chemical components, including VOCs and NVOCs, of DOH, DOL, DOF, and DOP. A total of 72 VOCs and 820 NVOCs were detected and identified using GC-MS and UPLC-MS/MS, respectively. The outcomes of the correlation analysis, PCA, and HCA collectively revealed substantial metabolic variations across DOH, DOL, DOF, and DOP, with particularly pronounced differences observed in DOH and others. DMs were annotated to six KEGG metabolic pathways, in which three mainly enriched pathways were found. *Trans*-nerolidol and 14 flavonoids were quantified using GC-MS and UPLC-DAD, respectively. DOH exhibited a significantly different level of *trans*-nerolidol and flavonoids compared to the other parts. Simultaneously, we compared the antioxidant activity among four *D. odorifera* parts. DOH, DOL, and DOF exhibited strong antioxidant capacity, suggesting that DOL and DOF have high potential for utilization. DOH showed significantly higher antioxidant capacity compared to the non-medicinal parts, and this was consistent with the results of component analysis. This study initially investigated the chemical composition and antioxidant activities of the non-medicinal parts (leaf, flower, and pod) of *D. odorifera*, aiming to evaluate their medicinal potential and promote the comprehensive utilization of this valuable species.

## Data Availability

Data are contained within the article and Supplementary Materials.

## Supplementary Materials

The following supporting information can be downloaded at: https://www.mdpi.com/article/10.3390/plants14213279/s1, Table S1: Identification and relative contents of VOCs in different parts of *D. odorifera*; Table S2: Identification of NVOCs in different parts of *D. odorifera*; Table S3: Key DMs annotated in the KEGG metabolic pathways. Table S4: Retention times (RTs) of 14 flavonoid standards. Table S5: Linearity, correlation coefficients, and sensitivity of 14 analytes determined by UPLC-DAD. Table S6: Precision, repeatability, stability, and recovery results (*n* = 6). Figure S1: The mass spectrometry fragment pathway of nerolidol oxide. Figure S2: Classical Venn diagram for VOCs (A) and NVOCs (B) in DOH, DOL, DOF and DOP. Figure S3: The GC-MS-SIM chromatograms of target compounds in four *D. odorifera* parts. Peak 1–5 represent nerolidol oxide I, II, *trans*-nerolidol, nerolidol oxide III, IV, respectively. Figure S4: The UPLC-DAD chromatograms of four *D. odorifera* parts at 275 nm. Peak 1–14 represent 3′,4′,7-trihydroxyisoflavone, butin, daidzein, liquiritigenin, luteolin, naringenin, tectorigenin, alpinetin, isoliquiritigenin, formononetin, 3’-O-methylviolanone, sativanone, pinocembrin, biochanin A, respectively.

## Abbreviations

The following abbreviations are used in this manuscript:

DOH	*Dalbergia odorifera* heartwood.
DOL	*Dalbergia odorifera* leaves.
DOF	*Dalbergia odorifera* flowers.
DOP	*Dalbergia odorifera* pods.
VOCs	Volatile organic compounds.
NVOCs	Non-volatile organic compounds.
