# Supplementary material for: Insights into Variations in Chemical Profiles and Antioxidant Properties Among Different Parts of Dalbergia odorifera
Source: Plants (Basel). 2025 Oct 27;14(21):3279. doi: 10.3390/plants14213279 (PMC12610685; doi:10.3390/plants14213279)
Supplement: Supplementary file 1 [file plants-14-03279-s001.zip › Supplementary Materials.pdf]

Supplementary Materials

Insights into Variations in Chemical Profiles and Antioxidant Properties Among Different Parts of *Dalbergia odorifera*

Yujie Xiao [1,2], YaKui Zhou [2], Jianhe Wei [1,2], Xiangsheng Zhao [2,*]

1 Institute of Medicinal Plant Development, Chinese Academy of Medical Sciences & Peking Union Medical College, Beijing 100193, China; xiaoyj0103@163.com (Y.X.); wjianh@263.net (J.W.)

2 Hainan Provincial Key Laboratory of Resources Conservation and Development of Southern Medicine, Hainan Branch of the Institute of Medicinal Plant Development, Chinese Academy of Medical Sciences & Peking Union Medical College, Haikou 570311, China; zhouyakui163@163.com

* Correspondence: xiangshengzhao@hotmail.com; Tel.: +86-898-31589013

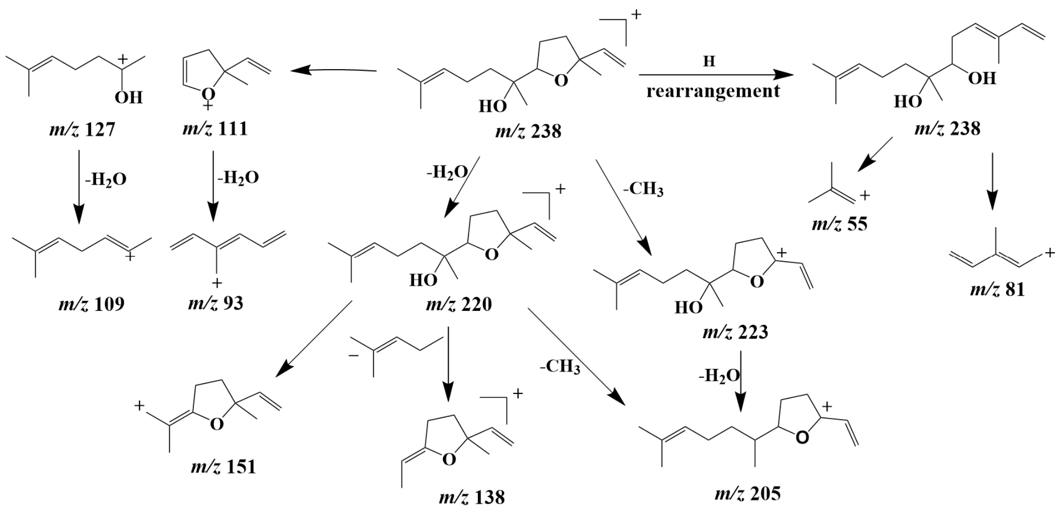

Figure S1. The mass spectrometry fragment pathway of nerolidol oxide.

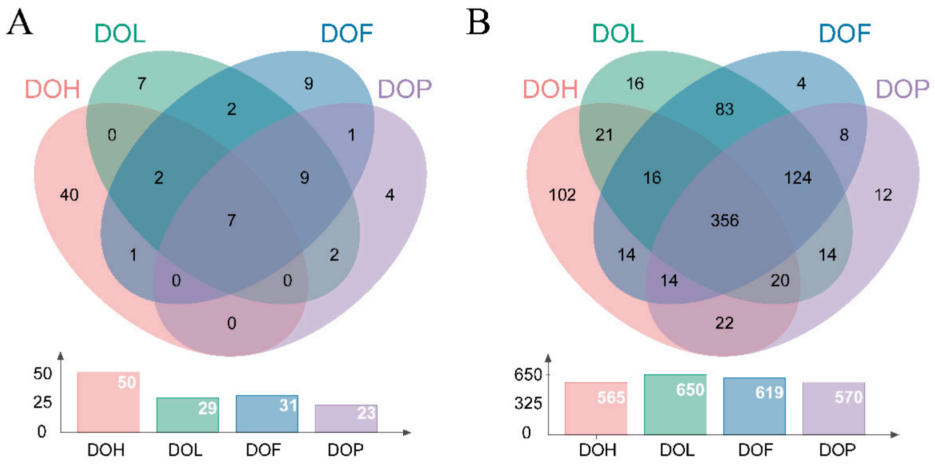

Figure S2. Classical Venn diagram for VOCs (A) and NVOCs (B) in DOH, DOL, DOF and DOP.

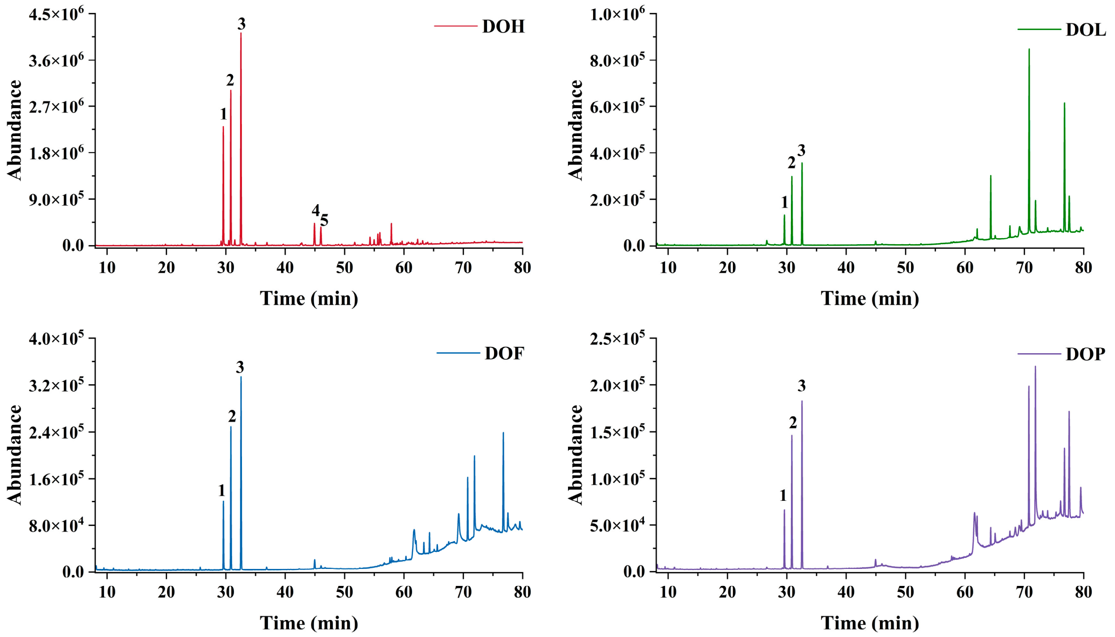

Figure S3. The GC-MS-SIM chromatograms of target compounds in four *D. odorifera* parts. Peak 1-5 represent nerolidol oxide I, II, *trans*-nerolidol, nerolidol oxide III, IV, respectively.

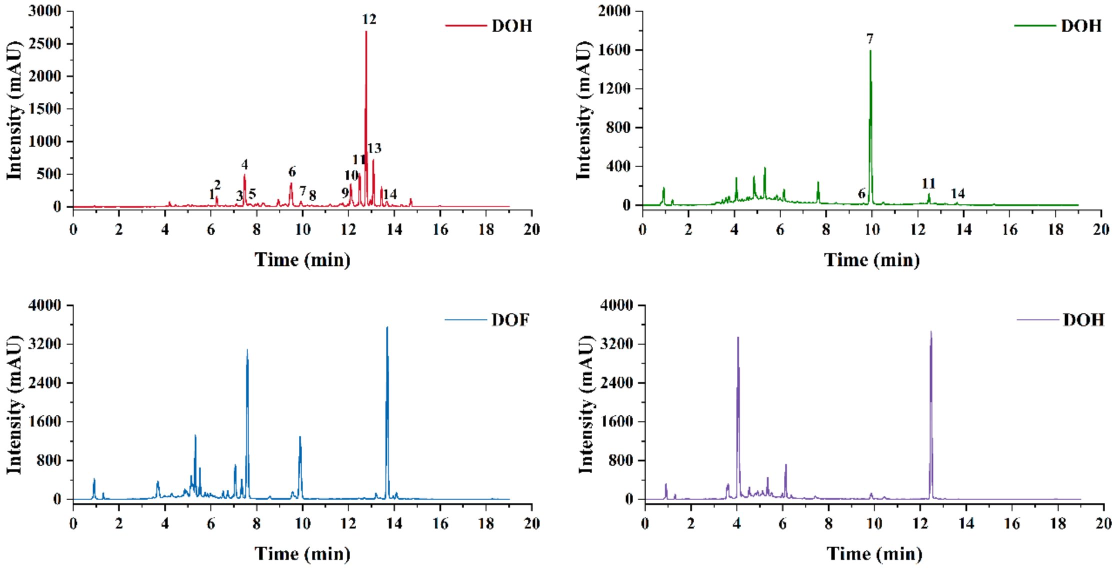

Figure S4. The UPLC-DAD chromatograms of four *D. odorifera* parts at 275 nm. Peak 1-14 represent 3′,4′,7-trihydroxyisoflavone, butin, daidzein, liquiritigenin, luteolin, naringenin, tectorigenin, alpinetin, isoliquiritigenin, formononetin, 3'-O-methylviolanone, sativanone, pinocembrin, biochanin A, respectively.

Table S4. Retention times (RTs) of 14 flavonoid standards.

Compound	Detection wavelength (nm)	Retention time (min)
3′,4′,7-Trihydroxyisoflavone	275	6.145
Liquiritigenin	275	6.303
Daidzein	275	7.291
Butin	275	7.543
Naringenin	275	7.815
Tectorigenin	275	9.717
Sativanone	275	10.049
Alpinetin	275	10.524
Isoliquiritigenin	360	11.844
Luteolin	360	12.192
3′-O-Methylviolanone	275	12.487
Formononetin	275	12.836
Pinocembrin	275	13.512
Biochanin A	275	13.772

Table S5. Linearity, correlation coefficients, and sensitivity of 14 analytes determined by UPLC-DAD

Analyte	Linear equation	R^2	Linear range ($\mu g \cdot mL^{-1}$)	LOD ($\mu g \cdot mL^{-1}$)	LOQ ($\mu g \cdot mL^{-1}$)
3′,4′,7-Trihydroxyisoflavone	Y=45.3782X−12.5724	0.9987	0.25–250	0.10	0.25
Butin	Y=50.8292X−0.8804	0.9991	0.25–750	0.10	0.25
Daidzein	Y=50.0671X−3.2139	0.9990	0.25–750	0.10	0.25
Liquiritigenin	Y=54.3307X−14.2311	0.9993	0.25–250	0.10	0.25
Luteolin	Y=35.8777X−42.2874	0.9977	0.5–250	0.20	0.50
Naringenin	Y=50.2647X−31.1170	0.9983	0.25–250	0.10	0.25
Tectorigenin	Y=63.1652X−8.3084	0.9989	0.25–750	0.10	0.25
Alpinetin	Y=40.7719X−1.0623	0.9991	0.6–300	0.20	0.60
Isoliquiritigenin	Y=100.9902X−7.7802	0.9991	1.25–375	0.50	1.25
Formononetin	Y=59.5289X−24.3469	0.9972	0.25–750	0.10	0.25
3′-O-Methylviolanone	Y=7.9522X−3.1243	0.9999	0.80–400	0.25	0.80
Sativanone	Y=45.202X+18.258	0.9998	0.25–200	0.10	0.25
Pinocembrin	Y=34.3467X+6.8163	0.9994	0.5–200	0.15	0.50
Biochanin A	Y=60.1673X+13.4684	0.9994	0.25–250	0.10	0.25

Table S6. Precision, repeatability, stability, and recovery results (n = 6)

Analyte	Intra-day RSD (%)	Inter-day RSD (%)	Repeatability RSD (%)	Stability RSD (%)	Recovery (%)
3′,4′,7-Trihydroxyisoflavone	0.19	0.82	1.77	2.14	96.48
Butin	0.19	1.28	2.32	0.16	97.07
Daidzein	0.17	1.34	3.52	3.77	96.99
Liquiritigenin	0.17	1.33	1.46	1.38	96.57
Luteolin	0.26	0.79	2.89	0.66	97.38
Naringenin	0.10	0.04	1.36	0.58	97.95
Tectorigenin	0.21	1.26	1.98	1.35	96.53
Alpinetin	0.17	1.19	3.14	2.84	98.22
Isoliquiritigenin	0.17	1.29	2.74	0.49	96.38
Formononetin	0.15	1.31	1.24	0.95	98.43
3′-O-Methylviolanone	0.84	1.12	1.20	1.38	97.58
Sativanone	0.33	1.84	3.22	1.49	96.26
Pinocembrin	0.28	0.92	1.71	0.99	95.15
Biochanin A	0.20	0.98	1.35	1.49	96.49
